# Mitochondria and Cancer

**DOI:** 10.1155/2013/763703

**Published:** 2013-01-30

**Authors:** Ryan Parr, Andrew Harbottle, John P. Jakupciak, Gurmit Singh

**Affiliations:** ^1^Mitomics Inc., Suite 1000, 290 Munro Street, Thunder Bay, ON, Canada P7A 7T1; ^2^Mitomics Inc., and Bioincubator Suite, Medical School, New Castle University, Framlington Place, Newcastle upon Tyne NE2 4HH, UK; ^3^Cipher Systems, Annapolis, MD 21401, USA; ^4^Juravinski Cancer Centre, 699 Concession Street, Hamilton, ON, Canada L8V 5C2; ^5^Department of Pathology and Molecular Medicine, McMaster University, 1280 Main Street W, Hamilton, ON, Canada L8N 3Z5

The future of mitochondrial DNA research has the potential to uncover new insights on genetic diseases and open new opportunities to discover ways to control mitochondria and their influence on the human health and cancer. The outcomes of this work will expand the understanding of cellular respiration and disease risk. In this special issue, we give examples of strategies for the measurement of oxidative stress; a critical factor in tumor progression. While the mitochondrial genome has been well characterized, the associations of the broad spectrum of mitochondrial genotypes remains a relatively rich field of study. 

Genetic tools are beginning to be realized which characterize mitochondrial populations and link their associations with normal and malignant cells. There are many large-scale deletions which require further investigation. There are populations of genotypes that rise and fall with tissue field effects. Mutations in the mitochondrial genome can alter the cellular biochemical behavior, changing the conditions for potential tumor growth. In this special issue we also address roles of mitochondrial interactions which are central to the physiological processes involved in malignant transformation. Measurements of DNA damage associated with prostate and other cancers can be normalized by comparative measurements of mitochondrial subpopulations. This can be used to assess DNA damage and somatic mutations under physiological and pathological conditions and can serve as a strategy to measure cell toxicity as a guide for devising innovative cancer preventions and treatments.

On account that mitochondrial DNA is accessible across various tissues, noninvasive collection and analyses are possible. From such investigations, it has been demonstrated that there is a progression of change in mitochondria through the tissues, as a field effect, that is, associated with the tumor tissue progression. Tumorigenic effects related to increasing ROS are well known in prostate and other solid tumor cancers. 

Mapping mutations across the entire mitochondrial genome are fundamental to the future work on mitochondrial “omic” investigations. Mitochondrial whole genome sequencing pioneered the concepts of conducting whole genome analyses to understand forensics. This has resulted in efforts to go beyond simple STR typing and to type the entire chromosome. It is because of the increased resolution achieved by sequencing whole metagenomes that, by extension, other fields of diagnostics, personalized medicine, and bacterial and viral forensics have emerged (J. P. Jakupciak unpublished data).

Through the study of mitochondria, mechanisms of cancer are emerging. The influence of mitochondria on the metastatic potential of cancer cell lines points to a promising future and in vivo characterization of populations of mitochondria will function as a “looking-glass” into monitoring the modulation events and even predicting changes in metastatic capabilities. Mitochondrial genomics is poised to enhance the over all field of omics and contribute significantly to the advent of personalized medicine [1].

In this special issue, the authors present some of the latest findings in this exciting and rapidly expanding area of genomic research:

- specific heteroplasmic somatic alterations in the mitochondrial genome contribute to the cell proliferation;
- a new paradigm for oxidative stress and cell and DNA damage has important implications for both cancer prevention and treatment;
- an assay for gauging systemic oxidative stress using peripheral blood;
- upregulation of a nuclear gene whose molecular interactions contribute to mitochondrial dysfunction, promoting cell proliferation;
- a cancerization field effect described by progressive mitochondrial mutations in noninvasiveand invasive breast cancer.

*Ryan Parr* *Andrew Harbottle* *John P. Jakupciak* *Gurmit Singh*
